# Dietary Patterns and Risk of Gallbladder Disease: A Hospital-based Case-Control Study in Adult Women

**Published:** 2015-03

**Authors:** Mahsa Jessri, Bahram Rashidkhani

**Affiliations:** ^1^Department of Nutritional Sciences, Faculty of Medicine, University of Toronto, Toronto, ON, Canada; ^2^Department of Community Nutrition Department, Faculty of Nutrition Sciences and Food Technology, National Nutrition and Food Technology Research Institute (WHO Collaborating Center), Shahid Beheshti University of Medical Sciences, Tehran, Iran

**Keywords:** Dietary patterns, Factor analysis, Gallbladder disease, Women, Iran

## Abstract

Gallbladder disease is one of the most prevalent gastrointestinal disorders that may result from a complex interaction of genetic and environmental factors. This study examined the association of dietary patterns with gallstone disease among Iranian women. This case-control study was conducted in general teaching hospitals in Tehran, Iran. Participants were 101 female cases and 204 female controls aged 40-65 years who were admitted for problems other than GBD. Dietary patterns were identified using principal components analysis based on food frequency questionnaire. Compared to the control group, cases were less educated, less physically active, and consumed more total energy (p<0.02). Having ≥3 livebirths increased the risk of gallstone by more than 5 times, followed by having rapid weight loss, being single, having familial history of gallstone, and consuming high total energy. Two distinct dietary patterns were identified in women (healthy and unhealthy). After adjustment for several confounding variables, healthy dietary pattern was associated with a decreased risk of gallstone disease (OR=0.14, 95% CI 0.048-0.4) while unhealthy dietary pattern was associated with an increased risk (OR=3.77, 95% CI 1.52-9.36). These findings confirm that dietary pattern approach provides potentially useful and relevant information on the relationship between diet and disease. Identifying risk factors will provide an opportunity for prevention of gallbladder disease in developing countries facing an increased risk of obesity.

## INTRODUCTION

Gallbladder disease (GBD) is the most common disorder of the biliary system, and most cases are asymptomatic (1). It is also one of the most common diseases affecting emergency-room patients with epigastric pain, nausea, vomiting, abdominal pain, and loss of appetite (2). Ten to 15 percent of Caucasian adults in developed countries have GBD (3). In a cross-sectional study in Iran, the prevalence of GBD in cadavers was 6.3% (4). Even though the prevalence in the Iranian men and women in the age-group of 31-40 years is low, it increases sharply in men older than 60 years and women older than 50 years to more than 10-fold (12.5 and 24.6% in males and females of 71-80 years respectively) (5). The pathogenesis of GBD is believed to be multifactorial and probably develops from interactions between several genetic and environmental factors (6,7).

Regarding diet as an important environmental factor, mounting evidence in nutritional epidemiology suggests that pattern analysis is the most realistic approach to assessing associations between overall diet and health or disease, instead of focusing on single dietary component (8). To our knowledge, only one study has addressed the relationship between dietary patterns and risk of GBD (9). There is a lack of published data in developing countries on this issue, and epidemiological studies in these countries could substantiate the existing evidence and provide valuable new information. The objectives of this study were to identify food patterns in Iranian women, using principal components analysis and to determine the association of certain dietary intake patterns with GBD.

## MATERIALS AND METHODS

### Study population

This case-control study was conducted in three teaching hospitals in Tehran city, (Taleghani, Shohadaye-Tajrish, and Shahid Modarress hospitals), Iran, from December 2009 to October 2010. Overall, 114 females (aged 40-65 years) admitted to these hospitals (with gallstone disease confirmed within the past 6 months of the interview) agreed to participate in this study (participation rate=83%). Controls were 214 hospital patients who were admitted to the same hospital as the cases, for a wide spectrum of diseases, such as orthopedic problems, ear/nose/throat diseases or elective surgeries and did not have history of dieting. The numbers of cases and controls were matched based on age (5-year groups) so that, in each age-group, the number of controls was twice as that of the cases. Totally, four cases and three controls were excluded from the analysis since their log scales of total energy intake was either >3 or <3 SD from the mean, indicating under/overreporting. After excluding the under- and overreporters for energy and those with missing data, 101 cases with gallstones and 204 controls were included in the analyses.

### Dietary assessment

Trained dietitians collected dietary data using a validated semi-quantitative food frequency questionnaire (FFQ) which assessed dietary intakes of cases one year before diagnosis and controls 1 year before interview. The FFQ consisted of 125 food items, including some of the most common Iranian meal recipes and has been previously shown to be valid and reproducible for use in Iranian adults (10,11). Consumption frequency was asked on a daily (e.g. bread), weekly (e.g. rice or meat) or monthly (e.g. fish) basis, and data from the questionnaire were transformed into the average monthly intake based on the assumption that each month is 30.5 days. For identifying the dietary patterns, 125 food items were grouped into 29 main food groups.

### Other measures

Using sets of prestructured pretested questionnaire, cases and controls were interviewed face-to-face during their hospital stay by trained professional interviewers. The questionnaire included information on sociodemographic characteristics, anthropometric variables, number of livebirths, physical activity, occupational history, familial history of gallstone, obesity and other diseases, history of rapid weight change, smoking status, and history of medication and supplement-use. Consumption of alcohol and opium was not responded in the present study due to subjects’ cultural and religious taboos. Physical activity was measured through interviews, using a previously-validated questionnaire (12). This questionnaire consisted of nine different metabolic equivalent (MET) categories on a scale ranging from sleep/rest (0.9) to high-intensity activities (>6). Multiplying the time spent in each activity by the MET value corresponding to that activity, MET.h for an activity was calculated. Crude MET.h/day was then calculated adding the MET.h values for different activities in a day. We further modified this value according to the under/overreporting status (compared to 24 hours in a day) so that the hours underreported were multiplied by 2 (the MET value for usual home activities) and were added to the crude physical activity value; the hours overreported were also multiplied by 2 and were subtracted from the crude physical activity value (13).

Weight was measured while subjects were standing on digital scales (Soehnle, Berlin, Germany) without shoes and was recorded to the nearest 100 g (14). Height was measured with subjects standing without shoes, using a non-stretch tape-meter fixed to a wall and was recorded to the nearest 0.5 cm. Weight was then divided by the square root of height for calculation of body mass index (BMI).

### Statistical methods

We used the ‘principal components method for factor analysis’ (15) of the Statistical Package for the Social Sciences software (version 16) (SPSS Inc., Chicago, IL, USA) to identify potential dietary patterns. Using the correlation matrix, the orthogonal varimax rotation method was applied to achieve a simpler structure facilitating interpretation. The number of meaningful components to retain from the total number of extracted patterns depended mainly on the assessment of scree plots and components’ interpretability (16). Each individual was then assigned a score according to the amount of food group consumption (in gramme) for each dietary pattern. This score was calculated according to the following formula:

j:i=Σj [(bij /λi)xj], where bij is the factor loading of food group j, xj is the dietary pattern i, λi is the eigen value for dietary pattern i and amount of food consumption is grammes for group j.

Factor loadings are correlation coefficients between food groups and dietary patterns; a positive loading in a factor indicates a direct association with the factor whereas a negative loading indicates that the food is inversely associated with the factor. Scores for two dietary patterns identified in this study (Factor 1 and 2) were then divided into two categories based on medians. We used chi-square tests to check the differences of distribution of categorical variables among cases and controls and for continuous variables; Student's *t*-test was used in case of normality, and Mann-Whitney test was used where the distribution of variables were not normal. Unconditional logistic regression was used in estimating odds ratio (OR) with 95% confidence interval (CI) after controlling for confounding variables (education, occupation, marital status, number of livebirths, physical activity, energy intake, familial history of gallstone, and history of rapid weight loss). All the reported p values are two-sided, and significance level is considered at <0.05.

### Ethics

The present study was approved by all regional ethics committees in Iran, and a written informed consent was obtained from each individual before the interview.

## RESULTS

Table 1 compares the characteristics of 101 cases and 204 hospital controls. By design, age was similar in both groups (53.54 years vs 52.91 years in cases and controls respectively). Compared to the controls, cases were significantly less educated (5.96 years vs 9.81 years of schooling, p<0.001), less physically active (38.62 MET.h/day vs 44.45 MET.h/day, p<0.001) and had more energy intake (2,661.2 kcal/day vs 2,433.4 kcal/day, p=0.027). Having ≥3 livebirths (OR=5.83, CI 3.43-9.9), having history of rapid weight loss (OR=2.85, CI 1.35-6.01), being single (OR=2.79, CI 1.30-6.01), having familial history of gallstone disease (OR=2.73, CI 1.65-4.52), and consuming ≥2,433 kcal/day (OR=1.28, CI 0.79-2.07) increased the risk of gallstones among participants. In contrast, having university education (OR=0.068, CI 0.029-0.16), having MET.h/day ≥40.58 (OR=0.35, CI 0.21-0.58), and having a job (OR=0.16, CI 0.05-0.53) decreased the risk of GBD (data not shown). Two dietary patterns were derived from the scree plot in factor analysis: (i) ‘healthy dietary pattern’ which included high amounts of vegetables, fruits, low-fat dairy products, vegetable oil, nuts, whole grains, legumes, fruit juice, fish and spices, and low intake of salt and (ii) unhealthy dietary pattern which comprised high intake of refined grain, tea, sugar, red meat, solid fat, soft drinks, baked potatoes, snacks, processed meats, high-fat dairy products, eggs, salt, pickled foods and sauerkraut and low intake of coffee. Coefficient alphas for the retained components are also shown in Table 2.

To highlight and compare the differences in dietary intakes below and above the median for each dietary pattern scores, mean daily intake (gramme/day) of selected foods are shown in Table 3. Mean intakes of nuts, fruits, vegetables, low-fat dairy products, fish, vegetable oil, whole grain, and red meat were significantly higher in the high category of healthy dietary pattern. In addition, soft drinks, sugar, low-fat dairy products, solid fat, refined grain, processed meat, and red meat were higher in the higher category of unhealthy dietary pattern.

Table 4 shows the distribution of some of the risk factors for gallstone disease according to the dietary pattern score medians. Increased healthy dietary pattern scores were associated with being employed (52.3% vs 37.3%, p<0.051) and not having familial history of gallstone (56.2% vs 38.2%, p<0.003). In addition, being in the highest category of unhealthy dietary pattern was associated with having ≥3 livebirths (61.5% vs 43.1%, p=0.003), being less educated (69.9% vs 34.5%, p<0.001) and being unemployed (53.5% vs 31.4%, p=0.004). Table 5 presents the OR and 95% CI for gallstone disease according to the dietary pattern categories. After adjustment for confounding variables, including education, occupation, marital status, number of livebirths, physical activity, energy intake, calcium supplement intake, familial history of gallstone and history of rapid weight loss, risk of gallstone disease among those with higher scores in healthy dietary pattern was 86% lower than those with lower scores (OR=0.14, 95% CI 0.048-0.4, p<0.001); while, in those with higher scores, risk of gallstone was 3.77 times higher compared to the first category (OR=3.77, 95% CI 1.52-9.36, p<0.004).

## DISCUSSION

In the present study, we identified two distinct dietary patterns among participants: healthy dietary pattern (which includes high intake of vegetables, fruits, fruit juice, low-fat dairy products, whole grain, nuts, legumes, and spices) and unhealthy dietary pattern (which includes high intake of processed meat, soft drinks, refined grains, red meat, high-fat dairy products, sugar, tea, solid fat, baked potato, snacks, egg, salt, pickled foods, and sauerkraut). Healthy dietary pattern appeared to be associated with the prevalence of decreased gallstone disease while unhealthy dietary pattern was associated with an increased risk.

**Table 1.** Characteristics of Iranian women in a case-control study of gallstone disease in 2009-2010, Tehran, Iran*,†

Variable	Participants		p value
	Case (N=101)	Control (N=204)	
Age (years)	53.54±5.8	52.91±7.8	0.431
Body mass index (kg/m^2^)	28.02±4.6	26.97±3.3	0.067
Waist-circumference (cm)	86.50±9.08	84.70±8.3	0.080
Education (years)	5.96±5.6	9.81±3.4	<0.001
Marital status			
Single	0 (0)	6 (2.9)	0.005
Married	84 (83.2)	182 (89.2)	
Divorced	0 (0)	3 (1.5)	
Widowed	17 (16.8)	13 (6.4)	
Menarche age (years)	13.05±1.54	13.56±1.49	0.123
Number of livebirths			
0	0 (0)	21 (10.3)	<0.001
1-3	39 (38.6)	140 (68.6)	
≥4	62 (61.4)	43 (21.1)	
Occupation			
Unemployed	96 (94.1)	168 (82.4)	<0.001
Employed	5 (4.9)	36 (17.6)	
Husband's occupation			
Unemployed	3 (3.5)	7 (3.9)
Grade 3 employee	45 (52.9)	68 (37.9)	0.048
Grade 2 employee	37 (43.5)	103 (57.5)	
Grade 1 employee	0 (0)	1 (0.56)	
Menopause age (years)	47.90±5.56	49.14±3.99	0.917
Physical activity (MET.h/day)	38.62±4.72	44.45±1.33	<0.001
Energy intake (kcal/day)	2,661.2±783	2,433.4±786	0.027
Familial obesity history			
Yes	45 (44.6)	83 (40.7)	0.6
No	56 (55.4)	121 (59.3)	
Familial history of gallstones			
Yes	50 (49.5)	55 (27)	<0.001
No	51 (50.5)	149 (73)	
Weight loss and regain history			
Yes	24 (25.2)	29 (14.5)	0.072
No	71 (74.7)	171 (85.5)	
Rapid weight loss history			
Yes	18 (17.8)	15 (7.4)	0.006
No	83 (82.2)	189 (92.6)	
Smoking habit			
Never	92 (91.1)	182 (91.5)	
Ex-smoker	2 (2)	8 (4.0)	0.807
Current-smoker	7 (6.9)	9 (4.5)	
Oral contraceptive usage			
Yes	27 (26.7)	54 (26.5)	0.991
No	74 (73.3)	150 (73.5)	
Exogenous hormone usage			
Yes	4 (4.1)	11 (5.5)	0.559
No	94 (95.9)	189 (94.5)	
Vitamin C supplement intake			
Yes	15 (15.2)	39 (19.5)	0.583
No	84 (84.8)	160 (80.4)	
Calcium supplement intake			
Yes	23 (23.4)	79 (39.1)	0.008
No	75 (76.5)	123 (60.8)	

*Values are presented either as mean±SD or n (%)

†Chi-square or Fisher's exact test was used for testing the difference

**Table 2.** Evaluation of food group factor loadings (correlation coefficients) of Iranian women in case-control study of gallstone disease in 2009-2010, Tehran, Iran*

Food groups	Healthy dietary pattern	Unhealthy dietary pattern
Vegetables	0.753	-
Fruits	0.640	-
Low-fat dairy products	0.549	-
Vegetable oil	0.468	-
Nuts	0.452	-
Whole grains	0.413	-
Legumes	0.388	-
High-fat dairy products	0.325	-
Fruit juice	0.2680	-
Fish	0.224	-
Spice	0.211	-
Refined grain	-	0.677
Tea	-	0.651
Sugar	-	0.614
Red meat	0.335	0.581
Solid fat	-	0.552
Soft drinks	-	0.425
Baked potatoes	-	0.371
Snacks	-	0.325
Processed meats	-	0.308
Coffee	-	-0.304
Egg	-	0.297
Pickles and sauerkraut	0.440	0.475
Salt	-0.308	0.280
Variance explained (%) coefficient alpha	13	9

*Values <0.2 are not shown for the purpose of simplicity

**Table 3.** Mean daily intake (grammes) of selected foods by median categories for each food intake pattern in case-control study of gallstone disease in 2009-2010, Tehran, Iran*

Dietary pattern	No.	Nuts	Fruits	Vegetables	Sweets and desserts	Soft drinks	Sugar	Low-fat dairy products	Fish	Solid fat	Vegetable oil	Refined grain	Whole grain	Processed meat	Red meat
Healthy														
Low	150	5.4±7.3	454.4±157.8	240.9±114.4	28.5±31.4	40.3±71.3	21.9±14.8	287.4±184.5	14.5±18.9	14.2±20.6	4.7±5.2	319.7±196.4	28.5±43.6	11.5±16.8	25.1±15.3
High	150	15.4±17	750.8±383.5	541.3±258.7	39.0±76.8	42.5±59.5	22.6±22.0	567.6±304.7	26.3±49.8	12.7±16.3	11.4±8.5	324.4±237.9	143.9±285.7	8.2±13.5	42.6±40.0
p value		<0.001	<0.001	<0.001	0.075	0.784	0.819	<0.001	0.004	0.489	<0.001	0.852	<0.001	0.073	<0.001
Unhealthy														
Low	152	9.7±12.6	635.2±364	388.6±249.8	29.6±73.2	17.4±10.9	13.5±11.7	388.9±245.0	22.7±51.5	6.5±6.59	8.85±8.28	218.6±128.4	106.5±271.3	7.34±13.8	24.2±25.0
High	148	11.4±14.3	612.8±296.0	401.8±295.6	39.5±24.9	66.9±67.6	31.7±21.5	468.9±245.0	18±20.6	20.6±13.5	7.2±7.15	425.4±244	63.5±95.2	12.6±14.5	41.6±37.5
p value		0.199	0.55	0.635	0.111	<0.001	<0.001	0.035	0.338	<0.001	0.069	<0.001	0.064	0.007	<0.001

*Low category comprises below median values, and high category corresponds to above median values

To our knowledge, Tseng *et al.*'s study is the only research evaluating the association of dietary patterns as identified by factor analysis with risk of GBD (17). Among 4,641 Mexican Americans aged 20-74 years, they detected four distinct patterns in women (vegetable, high calorie, traditional, and fruits) and three in men (vegetable, high calorie, and traditional). Only traditional dietary patterns in men was inversely associated with GBD (17).

Other studies, however, have assessed the role of individual dietary factors on the risk of GBD, and these suggest a more western diet, high in refined carbohydrates and fat (triglyceride) and low in fibre to induce the lithogenesis process (18). In the present study, healthy dietary pattern high in fruits, vegetables, whole grain, and vegetable oil was associated with decreased risk of GBD, which is in line with previous studies showing fruits and vegetables to have a protective role against gallstone disease through their high antioxidants (19), fibre, magnesium, and vitamin C content (20–22). Two previous studies on the role of vegetarian diet on gallstone disease suggest a protective role for vegetarian dietary pattern (23,24), and other studies show evidence for a protective effect of fruits (25–30) and vegetables (29), vegetable fat (31), vegetable protein (32), and crude fibre from vegetables (26), in the risk of GBD. In addition, vitamin C, folate, and magnesium have been linked to a lower risk of GBD, all of which are found profoundly in fruits and vegetables (33).

Consumption of sugar, refined grains, and soft drinks in the present study was higher in the unhealthy dietary pattern and was positively associated with an increased risk of gallstone disease. Generally, most of the previous studies revealed the consumption of refined sugar (34), dates (31), pastries, cakes (35), and beverages containing saccharose to be associated with increased risk of gallstone in both sexes (36). This could be attributed to higher cholesterol synthesis in the liver secondary to increased insulin in response to high sugar consumption (36). It has also been suggested that intake of 40 g of sugar per day doubles the risk of symptomatic gallstones (37) by inducing changes in metabolism of lipoproteins and, hence, modifications of the bile composition (38).

We also observed an increased risk of GBD with high intake of solid fat, red and processed meat, and egg within the unhealthy dietary pattern framework. Several studies have shown a higher total fat and saturated fat intake (39) among subjects with cholelithiasis (25) and suggest an aetiological role for these dietary components. Several studies have suggested that egg-yolk, red meat (29), animal fat (27), animal protein (19), and dietary cholesterol increase the biliary cholesterol saturation (40,41) and induce cholesterol gallstones (42,43), which is also confirmed in the present study.

**Table 4.** Distribution of selected risk factors of gallstone according to median of dietary pattern intake in a case-control study of gallstone disease among Iranian women in 2009-2010, Tehran, Iran*,†

Confounding variable	Level	Healthy dietary pattern		Unhealthy dietary pattern	
		Low No. (%)	High No. (%)	Low No. (%)	High No. (%)
Livebirths	<3	98 (52.1)	90 (47.9)	107 (56.9)	81 (43.1)
	≥3	50 (48.1)	54 (51.9)	40 (38.5)	64 (61.5)
	p value	0.507		0.003	
Physical activity, MET.h/day	<40.56	65 (47.1)	73 (52.9)	72 (52.5)	66 (50.6)
	≥40.56	85 (52.5)	77 (47.5)	80 (47.8)	82 (49.4)
	p value	0.354		0.63	
Education	Non-literate/Primary school	40 (54.8)	33 (45.2)	22 (30.1)	51 (69.9)
	Secondary school	28 (43.1)	37 (56.9)	30 (46.2)	35 (53.8)
	High school/Diploma	45 (46.9)	51 (53.1)	57 (59.4)	39 (40.6)
	University	32 (55.2)	26 (44.8)	38 (65.5)	20 (34.5)
	p value	0.407		<0.001	
Marital status	Married	132 (50.4)	130 (49.6)	130 (49.6)	132 (50.4)
	Single	15 (46.9)	17 (53.1)	17 (53.1)	15 (46.9)
	p value	0.708		0.708	
Occupation	Unemployed	116 (47.7)	127 (52.3)	113 (46.5)	130 (53.5)
	Employed	32 (62.7)	19 (37.3)	35 (68.6)	16 (31.4)
	p value	0.051		0.004	
Familial gallstone history	Yes	63 (61.8)	39 (38.2)	53 (52)	49 (48)
	No	84 (43.8)	108 (56.2)	95 (49.5)	97 (50.5)
	p value	0.003		0.685	
Rapid weight loss history	Yes	17 (53.1)	131 (49.6)	13 (40.6)	19 (59.4)
	No	15 (46.9)	133 (50.4)	136 (50.5)	128 (48.5)
	p value	0.708		0.245	

*Low category comprises below median values, and high category corresponds to above median values

†Values are n (%)

**Table 5.** Unadjusted and adjusted odds ratios (OR) and 95% confidence intervals (CI) for gallstone disease by median categories of food intake patterns in a case-control study among Iranian women in 2009-2010, Tehran, Iran*

Dietary pattern	Cases No. (%)	Controls No. (%)	OR (95% CI)†	Adjusted OR (95% CI)‡
Healthy				
Low	36 (36)	114 (57)	1.00 (Reference)	1.00 (Reference)
High	64 (64)	86 (43)	0.42 (0.259-0.696)	0.14 (0.048-0.4)
p value			0.001	<0.001
Unhealthy				
Low	74 (74)	74 (37)	1.00 (Reference)	1.00 (Reference)
High	26 (26)	126 (63)	4.85 (2.85-8.24)	3.77 (1.52-9.36)
p value			<0.001	0.004

*Low category comprises below median values, and high category corresponds to above median values

†This model is not adjusted for cofounding variables

‡Adjusted for confounding variables (education, occupation, marital status, number of livebirths, physical activity, energy intake, calcium supplement intake, familial history of gallstone, and history of rapid weight loss)

A protective effect has also been shown with fish oil and n-3 fatty acid consumption, which are suggested to lower the rate of cholelithiasis (44). In the present study, fish intake in the context of a healthy dietary pattern reduced the risk of GBD. This effect is in line with findings of some studies (30) and in contrast with a few others (45) which could be attributed to the ω-3 PUFA content of different types of fish. It has been shown that obese women on a weight-control diet, who take fish-oil supplements, will not experience cholesterol nucleation and gallstone disease as a result of weight loss compared to the control group (46). This confirms that ω-3 PUFA improves the cholesterol saturation index (CSI) and nucleation time and prevents cholelithiasis (46). In addition, changes in cholesterol metabolism could reduce the biliary cholesterol saturation and biliary protein concentration (47).

However, observed differences and contradictory results regarding the diet impact on the risk of GBD either are due to the specific factors in these foods or it might only represent the effect of other foods consumed in the diet (17). The contradiction in the information regarding the risk of specific dietary components on the risk of GBD could also be due to the ethnic differences, differences in lifestyle, and the difficulty of estimating specific dietary constitutes in individuals (48).

### Strengths and limitations

The present study had several strengths; the most important of all is being the first study to assess the association of dietary patterns with GBD in a developing country. Studies in developing countries can provide unique opportunities to test the association between diet and disease (49). In addition, where there is severe restriction in economic resources, food intake is strongly linked to income so that even small economic differences are directly reflected in diet which would increase the between-person variations. Furthermore, the different socioeconomic backgrounds of people in developing countries compared to majority of people in the western world (mainly the status of women, family-size, participation in work, autonomy, parity, and access to the healthcare systems) influence the outcome as a confounder.

In addition, the participation rate in this study was high (83%), which was another positive point. Subjects whose disease was diagnosed 6 months before the interview at the most were registered in the present study as incident cases in order to reduce the possibility of recall bias. This is due to the fact that dietary data collected at the time of disease ascertainment might not truly reflect past intakes or intakes during the development of disease. In addition, cases might have changed their diets due to the disease symptoms (protopathic bias). Controls were also carefully selected from patients only with conditions not related to diet or other major risk factors of GBD. Using the factor analysis is the third strength point of this study since the dietary patterns derived from factor analysis capture the effect of the combination of many interacting and synergic foods and represent a significant biological vision to focus on. This might result in stronger or more consistent results, which could be applied to various populations more easily. Compared to traditional analyses of single foods and nutrients, factor analysis has emerged as a method for comprehensive estimation of disease risk (50). In fact, complicated interactions among food items may not be studied adequately through a single-food approach, taking into account the high level of intercorrelation among some food items and the fact that the effect of a single food item may be too small to detect compared to the cumulative effect of multiple foods included in a dietary pattern (51). In addition, when food items are entered into a model simultaneously, the degree of independent variation of the food items is markedly reduced (52), making the examination of separate effects of dietary factors difficult. Dietary patterns are characterized as the habitual food intake and, hence, the collinearity of food items in factor analysis is used to advantage (51).

Before the implications of our findings are considered, it is necessary to consider potential limitations and biases. First, several arbitrary but important decisions are involved in factor analysis approach, including the labelling of the components, the number of factors to extract, the method of rotation and even their interpretation (53). Second, measurement errors were inevitable because dietary intake was assessed using FFQ which might have led to underestimation of associations. However, the FFQ used in this study has good validity and reproducibility among Iranian population (10,11), and we also excluded subjects who were under/overreporting their energy intakes. Third, recruitment of patients with other diseases as controls in the present study is a weakness since their exposure may not be representative of that in members of the study population who are at risk of becoming cases (54). In addition, control subjects might also have nutritional problems, which dilutes the association of dietary intakes and risk of gallstone disease due to sharing of the exposure. However, we prefered hospital controls (opposed to community controls) due to their higher participation and cooperation rates and to avoid selection bias. Furthermore, a range of control diagnoses was included to compensate for that limitation (54). Fourth, although residual confounding cannot be entirely ruled out due to imprecise measurements of important covariates, it is unlikely that errors in measuring the covariates were so extreme because the crude and multivariable results were essentially the same. Finally, it would have been ideal to match cases and controls for BMI values to be able to confidently compare these from a metabolic standpoint. In this study, we were concerned about overmatching problem which could lead to loss of efficiency since the matching effect could narrow the range of exposure.

### Conclusions

A healthy diet showed an inverse association with gallstone disease among Iranian women. However, case-control studies may prove an association but these do not demonstrate causation. As a result, these findings need to be confirmed in future prospective studies for aetiological purposes in both men and women to draw more conclusive results.

## ACKNOWLEDGEMENTS

Authors would like to thank the subjects for their enthusiastic support. We also thank the members of National Nutrition and Food Technology Research Institute for their collaboration. This work was financially supported by National Nutrition and Food Technology Research Institute of Shahid Behehshti University of Medical Sciences.

**Conflict of interest:** None of the authors had any personal or financial conflicts of interest.
